# Impact of Exposure to Intimate Partner Violence on CD4+ and CD8+ T Cell Decay in HIV Infected Women: Longitudinal Study

**DOI:** 10.1371/journal.pone.0122001

**Published:** 2015-03-27

**Authors:** Rachel Jewkes, Kristin Dunkle, Nwabisa Jama-Shai, Glenda Gray

**Affiliations:** 1 Gender & Health Research Unit, South African Medical Research Council, Pretoria, Gauteng, South Africa; 2 School of Public Health, University of the Witwatersrand, Gauteng, South Africa; 3 Office of the President, South African Medical Research Council, Western Cape, South Africa; Massachusetts General Hospital, UNITED STATES

## Abstract

Intimate partner violence (IPV) is a risk factor for HIV acquisition in many settings, but little is known about its impact on cellular immunity especially in HIV infected women, and if any impact differs according to the form of IPV. We tested hypotheses that exposure to IPV, non-partner rape, hunger, pregnancy, depression and substance abuse predicted change in CD4+ and CD8+ T-cell count in a dataset of 103 HIV infected young women aged 15-26 enrolled in a cluster randomised controlled trial. Multiple regression models were fitted to measure rate of change in CD4 and CD8 and including terms for age, person years of CD4+/CD8+ T-cell observation, HIV positivity at baseline, and stratum. Exposure variables included drug use, emotional, physical or sexual IPV exposure, non-partner rape, pregnancy and food insecurity. Mean CD4+ T cell count at baseline (or first HIV+ test) was 567.6 (range 1121-114). Participants were followed for an average of 1.3 years. The magnitude of change in CD4 T-cells was significantly associated with having ever experienced emotional abuse from a current partner at baseline or first HIV+ test (Coeff -132.9 95% CI -196.4, -69.4 p<0.0001) and drug use (Coeff -129.9 95% CI -238.7, -21.2 p=0.02). It was not associated with other measures. The change in CD8 T-cells was associated with having ever experienced emotional abuse at baseline or prior to the first HIV+ test (Coeff -178.4 95%CI -330.2, -26.5 p=0.02). In young ART-naive HIV positive women gender-based violence exposure in the form of emotional abuse is associated with a faster rate of decline in markers of cellular immunity. This highlights the importance of attending to emotional abuse when studying the physiological impact of IPV experience and the mechanisms of its impact on women’s health.

## Introduction

Intimate partner violence is recognised as a risk factor for HIV acquisition in many settings and there is good evidence to suggest that the pathways are substantially behavioural [1]. In the face of male violence, women are less able to utilise preventive practices, may acquiesce to male control in the relationship or alternatively are more likely to engage in risk behaviours [2]. There is also concern that violence exposure impacts on women’s immune system, either by rendering women more vulnerable to acquiring HIV or by enhancing disease progression after infection[3].

Evidence for the impact of violence on immunity is to date limited and unclear. There are some small studies have shown that women who experience violence have impaired humoural and cellular immunity, with elevated cortisol and dehydroepiandrosterone (DHEA) levels [4], and reduced T cell function[5], with the impact on cortisol mediated by the presence of PTSD in some studies[6] but not others[4]. A study has also shown association between C-reactive protein levels and PTSD in women with IPV exposure[7]. There has been no research on whether intimate partner violence is a risk factor for impaired cellular immunity in HIV positive women and whether it thus impacts on disease progression.

There is evidence that other social and biological factors impact on CD4 and these may confound any relationship between CD4 or CD8 and intimate partner violence. Depression and substance abuse are well recognised causes and consequences of intimate partner violence [8] and have also been associated with a faster rate of decline in CD4 in individuals with HIV. [9,10,11,12]. Pregnancy and food insecurity have also been shown to associated with a faster rate of CD4 decline [13,14], and since pregnancy is a well recognised period of risk from partner violence and food insecurity is a marker of poverty, which in general heightens partner violence risk, these were all considered to be important potential confounders. Exposure to child abuse has not been described in association with rate of CD4 decline in literature that we have been able to access, but it is plausible that there may be such an association.

This paper tests hypotheses that change in CD4 and CD8 T cell counts in a longitudinal dataset of HIV infected women who were part of the Stepping Stones study are associated with exposure to intimate partner violence. The data were collected between 2003–2006. When the study started anti-retroviral therapy (ART) was not available in the public health sector in South Africa. The policy to enable roll out was adopted in April 2004 but there was no availability in the study area until the very final stages of data collection. The population in this study was ART-naïve due to non-eligibility for ART based on CD4>200 and the slow roll out of ART in the public sector in the study area. The analysis presented here tests two hypotheses: i) that exposure to gender-based violence is associated with change in CD4 and CD8 indicating progression to HIV disease in young ART naïve women; and ii) that hunger, depression and substance abuse are associated with change in CD4 and CD8 as indicators of progression of HIV disease in young ART naïve women.

## Methods

Between 2002–2003 we enrolled youth into a cluster randomised controlled trial to evaluate the HIV prevention behavioural intervention Stepping Stones (25). Those eligible were aged 16–23 normally resident in the village where they were at school, and mature enough to understand the study and the consent process. At baseline, 1415 of the total enrolment were women. The focus of this paper is on the 89 women who were HIV infected at baseline and further 14 who had sero-converted by the 12 months follow up point and who all had two or more CD4 and CD8 tests during the study. The analysis only focuses on the period after HIV infection was established.

The trial had two study arms. One study arm received Stepping Stones, a 50 hour participatory intervention on sexual and reproductive health and HIV, delivered over 6–8 weeks. The control group received a 3 hour information session on safer sex and HIV, delivered on one occasion. In all other respects the participants in the two arms were treated no differently. They were volunteers recruited from schools in 70 locations (clusters) in the Eastern Cape province of South Africa. The clusters were divided into seven geographically-defined strata and equal numbers of clusters in each strata were randomised between the two study arms. In each cluster, 15–25 youth of each sex were enrolled. Baseline, 12 and 24 month assessments included a face-to-face questionnaire and blood was drawn for HIV and HSV2 testing. Those who tested HIV positive had CD4+ and CD8+ T cells measured at baseline and at months 12 and 24, except when blood was collected as a dried blood spot (only occurring for some women at 12 and 24 months follow up). The cohort was maintained using details collected at enrolment, with follow-up conducted nationwide as necessary to trace youth who had moved. Further information on all assessments, study recruitment, access and ethical issues, including support for participants testing HIV positive, is presented elsewhere (23, 26). Ethical approval for the study was given by the University of Pretoria. All participants gave written informed consent for participation in this research and records were de-identified prior to analysis. All were offered their HIV test results and offered psychological support from a study nurse. ART was available in the private sector during the study, but our funder did not permit use study resources for ART and so we could not provide it privately to our participants. However most of our HIV+ participants would not have been eligible for ART. Only 6 had a CD4<200 at diagnosis at baseline and the CD4 of one of these had risen to over 200 by the 12 month interview. At the 12 month follow up visit, a further 6 participants had CD4s T cells under 200. Thus a total of 12 would have been eligible for ART in the study. The pattern of CD4 T cell count of this group suggests that none were on ART as only one CD4 increased by >21 cells and this was from 114 to 262 over two years which does not suggest that ART was being taken.

### Measures

#### Biological

HIV-1 serostatus at baseline was assessed with two rapid tests performed in the site laboratory (27). The Determine (Abbott Diagnostics, Johannesburg) test was used for screening and specimens testing positive were retested with Uni-goldTM (Trinity Biotech, Dublin, Ireland). Indeterminate results were clarified using an HIV-1 screen ELISA (Genscreen) followed by two confirmatory ELISAs (Vironostika and Murex 1.2.0 if HIV positive). Towards the end of the second round of interviews collection of blood as dried spots was introduced for some participants to ease logistics and improve acceptability and these were sent to a central laboratory for testing. In the third round of interviews most blood was thus collected using the dry blood spot technique. The specimens were tested with a screen and confirmatory ELISA (as above). The CD4/CD8 T cell counts and ratios were determined by BD FACSOUNT analysis (BD Biosciences) in the National Institute for Communicable Diseases laboratories and blood samples were couriered to the lab for testing and storage. Blood was drawn for T cells counts at the time of the draw for the HIV test. Occasionally the sample was found not to be suitable for analysis and this explains some missing data for participants who had given blood samples potentially suitable for CD4/CD8 testing.

**Fig 1:**
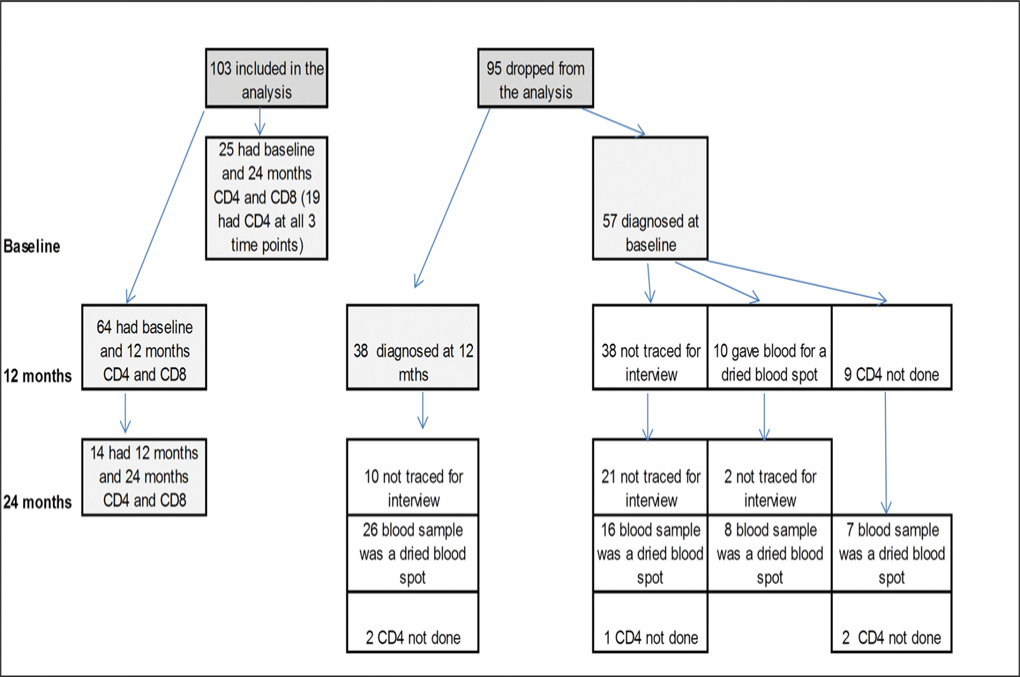
Flow Chart of Follow Up and Loss to Follow Up.

To derive a variable measuring change in CD4+ T cell counts we first deducted the CD4+ T cell count at 24 months from that available at baseline. We had measures at baseline and at 24 months for 25 participants (Fig. 1). For 64/198 participants we had CD4+ T cell count measured at baseline and 12 months follow up but not at 24 months, and for 14/198 participants we had CD4+ T cell counts at 12 months and 24 months. To measure CD4 decline, we first combined these assessments of CD4/CD8 change and then we set decline = 0 for participants in whom there was an increase in CD4 over the measured period. Thus the main variable modelled was a measure of CD4 decline. CD8 was handled in the same way. For each participant we calculated the person years of CD4/CD8 observation as the time between the first and last CD4.

#### Questionnaire and derived variables

We measured age and completed years of schooling. Food insecurity was measured at baseline by a question asking “Would you say that the people in your home often, sometimes, seldom or never go without food?” and was defined as responding ‘often’ as opposed to sometimes, seldom or never.

We measured alcohol use using the AUDIT scale which has been extensively validated in developing countries[15]. It has 12 items and asks about the frequency, quantity and consequences of drinking. We used a cut point of 8 for ‘problem drinking’[16]. We measured drug use by asking about ever use of dagga (marijuana), benzene, mandrax, injected drugs or other drugs and dichotomised respondents as those who had ever used drugs versus those who had not used drugs. Depression was measured with the Centre for Epidemiologic Studies Depression Scale (CES-D). The CES-D scale cut-off score of over 21 is regarded as indicating a high probability of clinical depression. The scale has a high sensitivity and specificity in otherwise well individuals (Weissman, Sholomskas, Pottenger, Prusoff, & Locke, 1977). We asked at if women had ever been pregnant at baseline and at each interview asked if they had had a pregnancy since the last interview. Adverse childhood experiences at baseline were measured on a modified version of the short form of the Childhood Trauma Questionnaire [17]. We assessed five dimensions of adversity: emotional neglect, emotional abuse, physical neglect/hardship, physical abuse and sexual abuse (Cronbach’s alpha for scale 0.77) and responses were added to create a score.

The WHO violence against women instrument was used to measure physical partner violence (5-items) and sexual partner violence (4 items) over the past year or ever (29). An affirmative response to any one of these questions was considered to have rendered the participant ‘exposed’. To derive a measure of greater severity, we coded physical and/or sexual violence into more than one episode versus none or only one, as this has been shown to be associated with multiple health outcomes (24). We also measured emotional abuse using five questions from the WHO violence against women instrument. These asked about whether a partner had insulted her, belittled or humiliated her in front of other people, done things to scare or intimidate her on purpose, threatened to hurt her, or stopped her from seeing any of her friends ever or in the last 12 months and we asked if it was her current partner who had done these things. Women were asked additional questions about experience of being ‘forced or persuaded to have sex against her will’ by a non-partner and experience of forced sex by more than one man (gang rape). These were combined in a measure of ever non-partner rape.

In order to examine the importance of contemporaneous violence during follow up, we derived variables for emotional abuse experience during the period of follow up and experience of more than one episode of physical or sexual intimate partner violence during the period offollow up. In each case we considered that a person had experienced these forms of violence if they disclosed any emotional abuse or more than one episode of physical or sexual violence during the period for which CD4/CD8 data was available.

### Data analysis

Analyses were carried out using Stata 12.0. There were 103/198 participants that had two CD4/CD8 measures and so were included in the analysis. Characteristics of participants followed up and not followed up were summarised as percentages (or means) with 95% confidence limits, using standard methods for estimating confidence intervals from complex multistage sample surveys (Taylor linearization). Pearson’s chi was used to test associations between categorical variables.

We examined a range of variables that may have been associated with a decline in CD4 or CD8 and summarised the mean decline and 95% confidence intervals. We used an appropriate regression model to provide the p value for each association. For this analysis a decline in CD4 was expressed as zero if there was no change or an increase.

To model factors associated with CD4 and CD8 change and take account for clustering of women within villages, random effects (multilevel) multiple regression models were fitted. These were built to measure the association between baseline (or pre-CD4 result, unless otherwise stated) exposures, which were potential factors which may impact on immunity. These included food security, alcohol and drug use, pregnancy, childhood trauma exposure and depression, and the outcomes of CD4 decline and CD8 decline. The models were built separately for CD4 and CD8 change, with all the potential associated factors entered into the model and then backwards elimination conducted to retain variables at p< or = 0.05. Each model retained age, CD4/CD8 years of follow up and HIV positivity at baseline (used as a crude indicator of duration of HIV infection) as adjustment variables. Models were tested for interactions and none were found.

## Results

103/198 women who tested HIV infected in the study had CD4+ and CD8+ T cells measured on two occasions and were included in the analysis. 95/198 HIV infected women did not have repeated CD4/CD8 measures and so were not included. The reasons for this are shown in Fig. 1. A comparison of the demographic, mental health and violence exposures and CD4s/CD8s of those retained in this analysis and those lost to follow up shows that they only differed in the proportion testing HIV positive for the first time at 12 months (Table 1). The proportion testing HIV+ initially at 12 months was higher among those with no subsequent CD4 follow up.

**Table 1:** Comparison of baseline characteristics of women who were HIV + at baseline, sero-convert at 12 months, and were retained in the analysis, and those lost to follow up.

	Followed up		Loss to follow up		
	n	%	n	%	p value
Age (mean)	103	19.6	95	19.4	0.266
Years of school at baseline: up to Grade 10	85	82.5	78	82.1	0.943
Grade 11	18	17.5	17	17.9	
Often hungry as without food	12	11.7	8	8.4	0.568
Depression	8	7.8	9	9.5	0.631
Alcohol problems	2	1.9	5	5.3	0.206
Ever used drugs	8	7.8	2	2.1	0.083
Ever emotional IPV	45	44.6	44	49.4	0.453
Ever physical IPV	42	40.8	47	51.1	0.202
Ever sexual IPV	19	18.6	16	17.6	0.843
>1 episode of physical or sexual IPV	35	34	34	35.8	0.813
Emotional abuse from current partner	28	27.7	23	25.8	0.77
HIV+ at baseline	91	88.4	57	60	<0.0001
sero-conversion at 12m	12	11.7	38	40	
cd4 at baseline (mean)	89	550.6	57	558.1	0.949
cd8 at baseline (mean)	89	1227.2	57	1377.6	0.085

Overall the CD4 mean was 567.6 (95%CI 523.5, 611.7) and the CD8 mean was 1218.2 (95%CI 1130.7, 1305.8). The mean number of years of observation was 1.31 (95% CI 1.22, 1.40). The overall mean change in CD4 was a decline of 42.2 cells/mm^3^ (95%CI -74.14399, -10.28319). The mean change in CD8 was a decline of 83.7 cells/mm^3^ (95%CI -161.8,-5.6). When examining purely CD4 decline, 42 women (40.8%) had no CD4 T cell decline, 8 (7.8%) had a small decline (≤ 20 cells/mm^3^) and 53 (51.4%) had larger declines (range 31–459). For CD8, 38 women (36.9%) had no decline, 4 had a small decline (<20) and 63 (59.2%) had larger declines (range 24–1048 cells/mm^3^). The mean CD4 decline was 84.19 cells/mm^3^ (95% CI 63.14, 105.24) and CD8 was 200.48 cells/mm^3^ (95% CI 152.21, 248.74).

Table 2 shows the unadjusted mean change in CD4/CD8 by hunger, mental health and violence exposures. There was no difference in mean change in CD4 across most exposures. Only having alcohol problems, having ever experience emotional abuse and emotional abuse by a current partner at baseline was associated with a significantly larger CD4 decline. The decline in CD8 was significantly larger only for women who had experienced physical partner violence, although at a 1% significance level exposure to hunger, drug use, > 1 episode of physical or sexual IPV during follow up, and having ever experienced emotional abuse may also have been associated.

**Table 2:** Mean decline in CD4 and CD8 by exposure variables (at timepoint prior to observed decline, unless otherwise stated).

	CD4 decline				CD8 decline			
	mean	95% CI		p value	mean	95% CI		p value
Years of school at baseline: up to Grade 10	91.5	69.3	113.8	0.066	211.2	155.7	266.8	0.284
Grade 11	49.5	5.9	93.1		149.6	56.3	242.9	
Often hungry as without food	103.5	45.0	162.0	0.450	344.3	146.9	541.8	0.082
Never or sometimes hungry	81.6	58.8	104.5		181.5	133.0	230.0	
Depression (CESD 16+)	132.9	19.4	246.4	0.258	129.6	-10.6	269.8	0.226
Not depressed	80.1	58.7	101.5		208.1	155.1	257.8	
Alcohol problems*	1.7	-58.7	8.8	<0.0001	173.3	-572.5	919.2	0.855
No alcohol problems	86.7	65.1	108.2		201.3	152.3	250.3	
Ever used drugs	132.6	43.1	222.1	0.212	338.5	111.5	565.5	0.104
No drug use	80.1	58.3	102.0		188.9	139.4	238.3	
Ever pregnant	67.3	37.1	97.4	0.221	184.8	100.6	269.0	0.693
Never pregnant	94.7	65.6	123.7		205.3	144.8	265.9	
Pregnant during follow up	66.1	24.3	108.0	0.316	177.1	44.4	309.8	0.645
Not pregnant during follow up	89.1	64.5	113.7		206.8	155.4	258.2	
Ever emotional IPV	107.9	77.8	138.1	0.047	256.4	176.6	336.3	0.096
No emotional abuse	59.1	32.8	85.4		156.2	96.1	216.3	
Emotional abuse by current partner	134.2	93.1	175.4	0.003	254.8	165.2	344.5	0.214
None	60.0	38.5	81.5		180.5	121.3	239.8	
Ever physical IPV	95.7	69.7	121.7	0.297	256.9	180.6	333.1	0.038
No physical IPV	76.0	44.5	107.4		160.1	98.0	222.2	
Ever sexual IPV	91.8	45.5	138.1	0.695	224.6	115.1	334.0	0.557
No sexual IPV	83.5	59.2	107.7		197.1	141.9	252.4	
>1 episode of physical or sexual IPV	98.4	67.3	129.4	0.277	263.6	173.2	354.0	0.078
1 or no P/S IPV	76.9	48.9	104.9		168.0	111.5	224.5	
Rape by a non-partner	80.9	4.7	157.0	0.903	322.2	34.3	610.1	0.309
No non-partner rape	84.5	62.2	106.8		188.8	141.6	236.1	
>1 episode of physical or sexual IPV during follow up	98.0	58.4	137.6	0.907	263.3	155.2	371.5	0.104
1 or none	79.0	53.7	104.3		177.0	123.7	230.3	
Any emotional abuse during follow up	90.3	61.3	119.2	0.506	235.3	163.5	307.2	0.180
none	78.0	46.5	109.5		164.9	99.6	230.3	

(*NB small numbers n = 2)

Table 3 shows the multi-variable regression models of factors associated with CD4 or CD8 change. It shows that having ever emotional abuse from a current partner was significantly associated having greater CD4 change (Coeff -132.9 95% CI -196.4, -69.4 p<0.0001), and having ever experienced emotional abuse was associated with a significant change in CD8 (Coeff -157.8 95% CI -313.5, -2.14 p = 0.047). The models also showed that that having ever used drugs was associated with a decline in CD4. The direction of these changes was such that in each case they increased the rate of decline in CD4 and CD8 counts. Neither depression, pregnancy, child abuse, hunger nor alcohol were associated with a decline in either cellular immunity marker. None of the other measures of violence were associated with CD4 or CD8 change.

**Table 3:** Multivariable models of social factors, mental health and partner violence exposures associated with change in CD4/CD8 (models adjusted for age, CD4/8 follow up period, HIV positivity at baseline.

	CD4 change*				CD8 change**			
	Coefficient	95% CI		P value	Coefficient	95% CI		P value
Experienced emotional abuse from current partner	-132.87	-196.37	-69.37	<0.0001				
Ever experienced emotional abuse					-178.37	-330.24	-26.49	0.02
Ever used drugs	-129.90	-238.68	-21.22	0.019				

* Wald chi square p = 0.0002

** Wald chi square p = 0.04

## Discussion

In this analysis we tested two hypotheses. With respect to the first hypothesis, we have shown that exposure to emotional abuse is a risk factor for faster decline in CD4 and CD8 in HIV infected young women, after adjustment for available indicators of duration of HIV disease and other risk factors. We did not show that exposure to physical or sexual intimate partner violence was associated with rate of change in CD4 or CD8, although there was a suggestion of this for CD8 in the bi-variable analysis. In respect of the second one, we showed that drug use was associated with a more rapid decline in CD4 in HIV infected young women. In contrast to previous literature and samples, we did not show that the other variables were associated with CD4 decline, although there was a suggestion of a marginal effect for food insecurity.

In the models the coefficients can be interpreted as reflecting the impact on change in CD4 and CD8 in cells/mm^3^ among those with emotional abuse exposure when compared to the unexposed. Given that the period of observation of on average 1.3 years, the adjusted mean change in CD4 among those with emotional abuse exposure was 102 cells/mm^3^ per annum greater than those without emotional abuse exposure. Research from Europe suggests that in the absence of ART CD4 declines at a rate of 50–70 cells/mm^3^ per annum [18,19]. This suggests that the impact of emotional abuse exposure is to increase this rate by 50–100%. With the mean CD4 of 567 at baseline 9or HIV+ diagnosis), and with anti-retroviral therapy (ART) commencing in South Africa at 350 cells/mm^3^, this increase in rate would have considerably accelerated ART initiation in the emotional abuse exposed group.

Emotional abuse has generally received little attention from programmes, but there is a growing body of evidence to support its importance as a risk factor for ill health in some populations. There is good evidence of it being a risk factor for post-natal depression [20], and some evidence from the dataset analysed for this paper that experience of emotional abuse alone was associated with suicidality, and in conjunction with physical and sexual violence it was associated with depression, drug use and alcohol abuse [21]. This analysis shows that it had an important impact on cellular immunity on these young HIV infected women, that could not be explained by depression or other aspects of the mental health impact of emotional abuse, nor by co-exposure to physical or sexual violence. It would be useful to explore mediating pathways in future research, which may include impact on self-esteem and perceptions of power.

There has been interest in whether IPV exposure enhances susceptibility to HIV[3]. Our data does not directly contribute to this debate as we have no information on the hormonal or cellular immunity of those who were HIV negative and acquired HIV during the study prior to their testing HIV positive However we have shown that there is an impact on immunity of IPV exposure among HIV positive women, but it seems to be an impact of emotional abuse.

This study has the advantage of being longitudinal and conducted in an untreated HIV infected+ population, which enabled an analysis which would otherwise not have been possible. We acknowledge the limitations of the small sample size. We did not have information on opportunistic infections, but expect that if these varied during the follow up period according to intimate partner violence baseline exposures it would be due to the impact of such exposure on immunity. True loss to follow up in this study group was limited, however many of the women who had baseline T cell counts did not have a subsequent one for reasons which were (mostly due to study related decisions or omissions. Only a third (n = 33) of the 95 women who were not followed up were lost due to participant related factors. Loss to follow up does not seem to have been differential according to CD4 or CD8 or violence exposures. Levels of violence exposure reporting were high, which suggests that there was minimal under-ascertainment. We are aware of a debate about the importance of CD4 and CD8 ratios and assertions that in the absence of ART those HIV infected+ persons with a declining CD4 have an increasing CD8 count[22]. However in this dataset 78.5% of persons with a measured CD4 decrease (of > 10 counts) had a CD8 decrease (of > 10) during the same period of observation.

## Conclusions

We have shown that in a sample of young ART-naive HIV positive women, gender-based violence exposure in the form of emotional abuse was associated with a faster rate of decline in markers of cellular immunity and progression of HIV disease. This study is one of the first to provide evidence of an impact of gender-based violence on cellular immunity in HIV positive women. It highlights the importance of attending to emotional abuse when studying the physiological impact of intimate partner violence experience and the mechanisms of its impact on women’s health.

## References

[1] Jewkes R, Dunkle K, Nduna M, Shai N. Intimate partner violence, relationship gender power inequity, and incidence of HIV infection in young women in South Africa: a cohort study. The Lancet 2010; 367: 41–48. 10.1016/S0140-6736(10)60548-X 20557928

[2] Jewkes R. Gender inequities must be addressed in HIV prevention. Science 2010; 329: 145–147. 10.1126/science.1193794 20616253

[3] Campbell JC, Baly ML, Ghandour RM, Stockman JK, Francisco L, Wagman J. The intersection of intimate partner violence against women and HIV/AIDS: a review. Int J Inj Contr Saf Promot 2008; 15: 221–231. 10.1080/17457300802423224 19051085 PMC3274697

[4] Pico-Alfonso MA, Garcia-Linares MI, Celda-Navarro N, Herbert J, Martinez M. Changes in cortisol and dehydroepiandrosterone in women victims of physical and psychological intimate partner violence. Biol Psychiatry 2004; 56: 233–240. 15312810 10.1016/j.biopsych.2004.06.001

[5] Constantino RE, Sekula LK, Rabin B, Stone C. Negative life experiences, depression, and immune function in abused and nonabused women. Biol Res Nurs 2000; 1: 190–198. 11232214 10.1177/109980040000100304

[6] Inslicht SS, Marmar CR, Neylan TC, Metzler TJ, Hart SL, Otte C, et al Increased cortisol in women with intimate partner violence-related posttraumatic stress disorder. Psychoneuroendocrimology 2006; 31: 825–838. 10.1016/j.psyneuen.2006.03.007 16716530

[7] Heath NM, Chesney SA, Gerhart JI, Goldsmith RE, Luborsky JL, Stevens NR, et al Interpersonal violence, PTSD, and inflammation: potential psychogenic pathways to higher C-reactive protein levels. Cytokine 2013; 63: 172–178. 10.1016/j.cyto.2013.04.030 23701836 PMC3731749

[8] World Health Organisation, London School of Hygiene and Tropical Medicine. Preventing intimate partner and sexual violence against women: taking action and generating evidence. Geneva: World Health Organisation 2010 10.1136/ip.2010.029629

[9] Burack JH, Barrett DC, Stall RD, Chesney MA, Ekstrand ML, Coates TJ. Depressive symptoms and CD4 lymphocyte decline among HIV-infected men. JAMA 1993; 270: 2568–2573. 7901433

[10] Baum MK, Rafie C, Lai S, Sales S, Page B, Campa A. Crack-cocaine use accelerates HIV disease progression in a cohort of HIV-positive drug users. J Acquir Immune Defic Syndr 2009; 50: 93–99. 10.1097/QAI.0b013e3181900129 19295339

[11] Baum MK, Rafie C, Lai S, Sales S, Page JB, Campa A. Alcohol use accelerates HIV disease progression. AIDS Res Hum Retroviruses 2010; 26: 511–518. 10.1089/aid.2009.0211 20455765 PMC2875959

[12] Meijerink H, Wisaksana R, Iskandar S, den Heijer M, van der Ven AJ, Alisjahbana B, et al Injecting drug use is associated with a more rapid CD4 cell decline among treatment naive HIV-positive patients in Indonesia. J Int AIDS Soc 2014; 17: 18844 10.7448/IAS.17.1.18844 24388495 PMC3880941

[13] Lieve V, Shafer LA, Mayanja BN, Whitworth JA, Grosskurth H. Effect of pregnancy on HIV disease progression and survival among women in rural Uganda. Trop Med Int Health 2007; 12: 920–928. 17697086 10.1111/j.1365-3156.2007.001873.x

[14] McMahon JH, Wanke CA, Elliott JH, Skinner S, Tang AM. Repeated assessments of food security predict CD4 change in the setting of antiretroviral therapy. J Acquir Immune Defic Syndr 2011; 58: 60–63. 10.1097/QAI.0b013e318227f8dd 21694604 PMC3159819

[15] Kaner EF, Dickinson HO, Beyer F, Pienaar E, Schlesinger C, Campbell F, et al The effectiveness of brief alcohol interventions in primary care settings: a systematic review. Drug Alcohol Rev 2009; 28: 301–323. 10.1111/j.1465-3362.2009.00071.x 19489992

[16] Saunders JB, Aasland O, Babor T, de la Fuente JR, Grant M. Development of the Alcohol Use Disorders identification test (AUDIT): WHO Collaborative project on early detection of persons with harmful alcohol consumption II. Addiction 1993; 88: 791–804. 8329970 10.1111/j.1360-0443.1993.tb02093.x

[17] Bernstein DP, Fink L, Handelsman L, Foote J, Lovejoy M, Wenzel K, et al Initial reliability and validity of a new retrospective measure of child abuse and neglect. American Journal of Psychiatry 1994; 151: 1132–1136. 8037246 10.1176/ajp.151.8.1132

[18] Lodi S, Phillips A, Touloumi G, Pantazis N, Bucher HC, Babiker A, et al CD4 decline in seroconverter and seroprevalent individuals in the precombination of antiretroviral therapy era. AIDS 2010; 24: 2697–2704. 10.1097/QAD.0b013e32833ef6c4 20885283

[19] Touloumi G, Pantazis N, Pillay D, Paraskevis D, Chaix ML, Bucher HC, et al Impact of HIV-1 subtype on CD4 count at HIV seroconversion, rate of decline, and viral load set point in European seroconverter cohorts. Clin Infect Dis 2013; 56: 888–897. 10.1093/cid/cis1000 23223594

[20] Ludermir AB, Lewis G, Valonguiero SA, Barreto de Araujo TV, Araya R. Violence against women by their intimate partner during pregnancy and postnatal depression: a prospective cohort study. The Lancet 2012; 376: 903–910. 10.1016/S0140-6736(10)60887-2 20822809

[21] Jina R, Jewkes R, Hoffman S, Dunkle K, Nduna M, Jama-Shai N. Adverse health outcomes associated with emotional abuse in young rural South African women: a cross-sectional study. Journal Interpersonal Violence 2012; 27: 862–880 10.1177/0886260511423247 21987516 PMC3581304

[22] Margolick JB, Muñoz A, Donnenberg AD, Park LP, Galai N, Giorgi NV, et al Failure of T-cell homeostasis preceding AIDS in HIV-1 infection. The Multicenter AIDS Cohort Study. Nat Med 1995; 1: 674–680. 7585150 10.1038/nm0795-674

